# Supplementary material for: MetaRibo-Seq measures translation in microbiomes
Source: Nat Commun. 2020 Jun 29;11:3268. doi: 10.1038/s41467-020-17081-z (PMC7324362; doi:10.1038/s41467-020-17081-z)
Supplement: Supplementary file 10 — Supplementary Data 7 [file 41467_2020_17081_MOESM10_ESM.zip › File2/Confidence_VeryHigh_Taxonomy/109757_out.krona.html]

Javascript must be enabled to view this page.

members
magnitude
magnitudeUnassigned
count
unassigned
taxon
rank

109757\_out

1
6

SRS023914\_contig\_number\_4302

3

SRS013687\_contig\_number\_contig-100\_9193.129906SRS015190\_contig\_number\_contig-100\_2370.43998SRS019685\_contig\_number\_25368

2
superkingdom
2

phylum
2
1224

2
class
1236

1775403
order
2

568386
family
2

413435
2
genus

2303331
species
2

SRS053356\_contig\_number\_23613SRS1041145\_contig\_number\_contig-100\_1828.145799
